# Bioinspired Underwater Superoleophobic Microlens Array With Remarkable Oil-Repellent and Self-Cleaning Ability

**DOI:** 10.3389/fchem.2020.00687

**Published:** 2020-08-04

**Authors:** Hao Bian, Jie Liang, Minjing Li, Fan Zhang, Yang Wei

**Affiliations:** ^1^School of Electronic Science and Engineering, Xi'an Jiaotong University, Xi'an, China; ^2^Wuhan National Laboratory for Optoelectronics, Huazhong University of Science and Technology, Wuhan, China; ^3^School of Mechanical Engineering, Xi'an Jiaotong University, Xi'an, Shaanxi, China

**Keywords:** microlens array, underwater superoleophobic, anti-oil, self-cleaning, femtosecond laser microfabrication

## Abstract

Underwater superoleophobic microlens array (MLA) has been emerging as a crucial device for its wide applications in ocean optical imaging and sensing, endoscopic surgery, microfluidics and optofluidics, and other biomedical applications. Fabrication of microlens arrays integrated with excellent optical performance as well as underwater superoleophobicity remains a great challenge. In this paper, we report an underwater super oil-repellent MLA on a transparent optical glass substrate via femtosecond laser-induced phase and structural modification and chemical isotropic etching. The fabricated sample simultaneously possesses microlens structures with a smooth surface to enable optical imaging function, and grid-patterned biomimetic micro/nano hierarchical surface structures to produce underwater oil-resistance with a contact angle of 160.0° and a sliding angle of 1.5°. The resultant oil-repellent MLA exhibits underwater superoleophobicity and self-cleaning abilities in water. Meanwhile, it was demonstrated to have impressive imaging capability even after oil contamination. We believe that this novel resultant anti-oil MLA will be helpful for underwater detection and bioscience research, especially in oil polluted underwater workspaces.

**Graphical Abstract:**
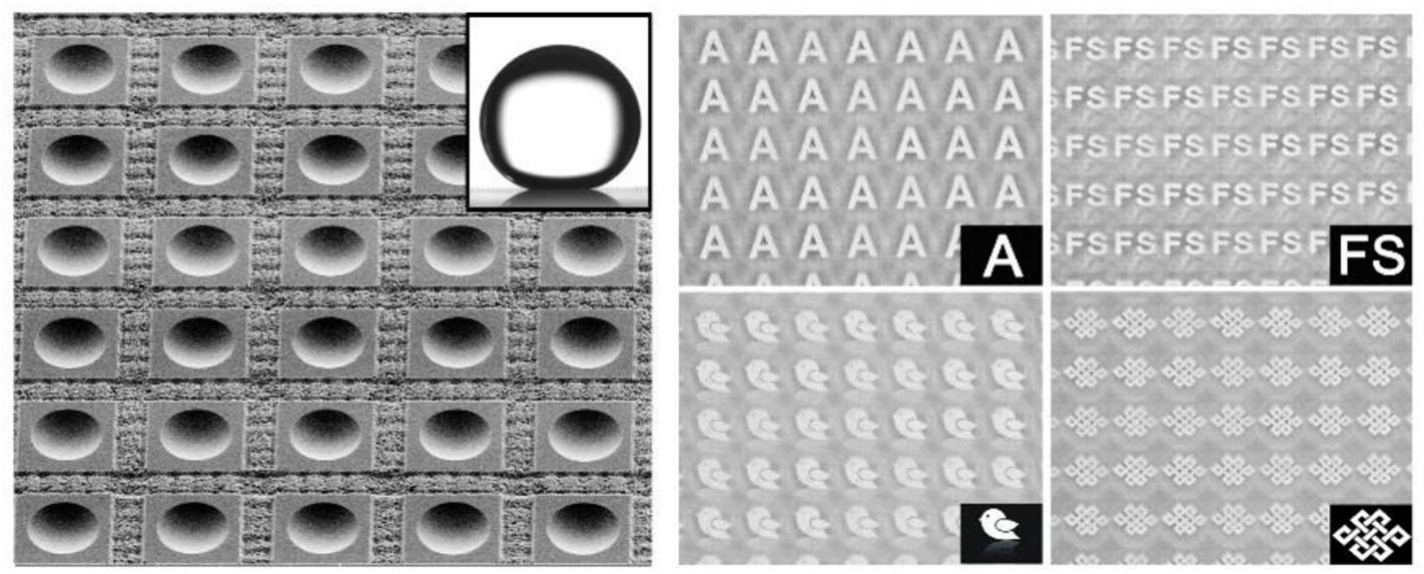
SEM image of the underwater superoleophobic microlens array and its imaging performance.

## Introduction

Underwater optical detection attracts enormous interests in ocean exploration and exploitation, endoscopic surgery, lab-on-chip devices, and many other bioscience applications (Dong et al., 2007; Barretto et al., 2009; Fei et al., 2011; Wu et al., 2015; Vespini et al., 2016). The MLA, as a crucial component for compact, integrated and miniaturized optical system, can be used for direct microimaging and data collection during underwater optical detection (Krupenkin et al., 2003; Li et al., 2016a,b; Yang et al., 2016; Zhang et al., 2017). Various methods were presented to fabricate MLA (Chan and Crosby, 2006; Lee et al., 2012; Jiang et al., 2014; Wu et al., 2014; Zhang et al., 2014; Jung and Jeong, 2015; Yong et al., 2015a). However, conventional MLAs with smooth surface structures cannot withstand liquid contaminates, such as oils and other organic solutions. Such pollution may hinder their sustained use and degrade optical properties. More specifically, the microlenses used for imaging during endoscopic surgery, which involves blood vessels, intestines, and stomach, can be easily polluted by oils and other organic solutions which will seriously weaken the optical performance. The main strategies for the underwater oil-repellent MLA rely on an additional optical protection window or chemical coating for micro-optical system which suffers the complex configuration, unsustainable, and potential chemical pollution. Therefore, fabrication of integrated oil-repellent MLA for underwater detection still remains a huge challenge.

Many natural surfaces exhibit underwater superoleophobicity and self-cleaning capability, such as fish scales (Liu et al., 2009; Cai et al., 2014), clam's shell (Liu et al., 2012), seaweed (Chen et al., 2015) < etc. (Lin et al., 2010; Nishimoto and Bhushan, 2013; Xu et al., 2013; Wang et al., 2015). Mimicking these natural creatures, many fabrication methods, such as self-assembly (Cheng et al., 2014, 2015), template methods (Cheng et al., 2011), dipping coating, etc. (Li et al., 2015, 2016c; Liu et al., 2015), were used to achieve underwater superoleophobic surfaces which generally combine the hydrophilic chemical composition and the micro/nano hierarchical rough surface topography. For instance, inspired by a short clam's shell, Jiang et al. imitated an artificial inorganic high-energy copper oxide coating on a metal copper sheet, which shows underwater superoleophobicity and self-cleaning capability (Lin et al., 2010). Inspired by the Diphylleia grayi's petals, Yong et al. fabricated a transparent underwater superoleophobic surface on silica glass by femtosecond laser ablation. Such rough surfaces exhibited high transparency, underwater superoleophobicity, and oil-resistance in water (Yong et al., 2015b). How to endow MLAs with these biomimetic superoleophobic structures especially on engineering applied materials deserves further study.

Femtosecond laser microfabrication has demonstrated to be an effective way to fabricate MLA and hierarchal micro/nano rough structure. Benefit from the extreme non-linear phenomenon of the interaction between light and material, femtosecond laser was adopted to realize many very unique structures and devices which nanosecond laser or picosecond laser is insufficient (Gattass and Mazur, 2008). The microlens structure and MLAs were successfully obtained utilizing femtosecond laser enhanced chemical isotropic etching process (Hao et al., 2012; Chen et al., 2020). The hierarchical micro/nano surface structures were also achieved via femtosecond laser direct writing which inspired numerous researchers to realize surfaces with superwettability (Zhang et al., 2012; Yong et al., 2013, 2020; Yin et al., 2017; Duan et al., 2018; Bai et al., 2020; Wu et al., 2020). Here, we present a simple way to fabricate a novel anti-oil MLA on a glass substrate by femtosecond laser-induced chemical isotropic etching and selective direct laser ablation (DLA) processes, respectively. In detail, the as-prepared sample possesses smooth microlenses which were formed by femtosecond laser enhanced chemical etching are used for optical imaging. Besides, the surrounding grid-patterned hierarchical micro/nano rough structures, which were created by DLA, endow the sample underwater superoleophobicity. Not only will the anti-oil MLA exhibit excellent underwater optical performance, but it also has anti-oil and self-cleaning capacity.

## Experimental Section

**Figure 1:**
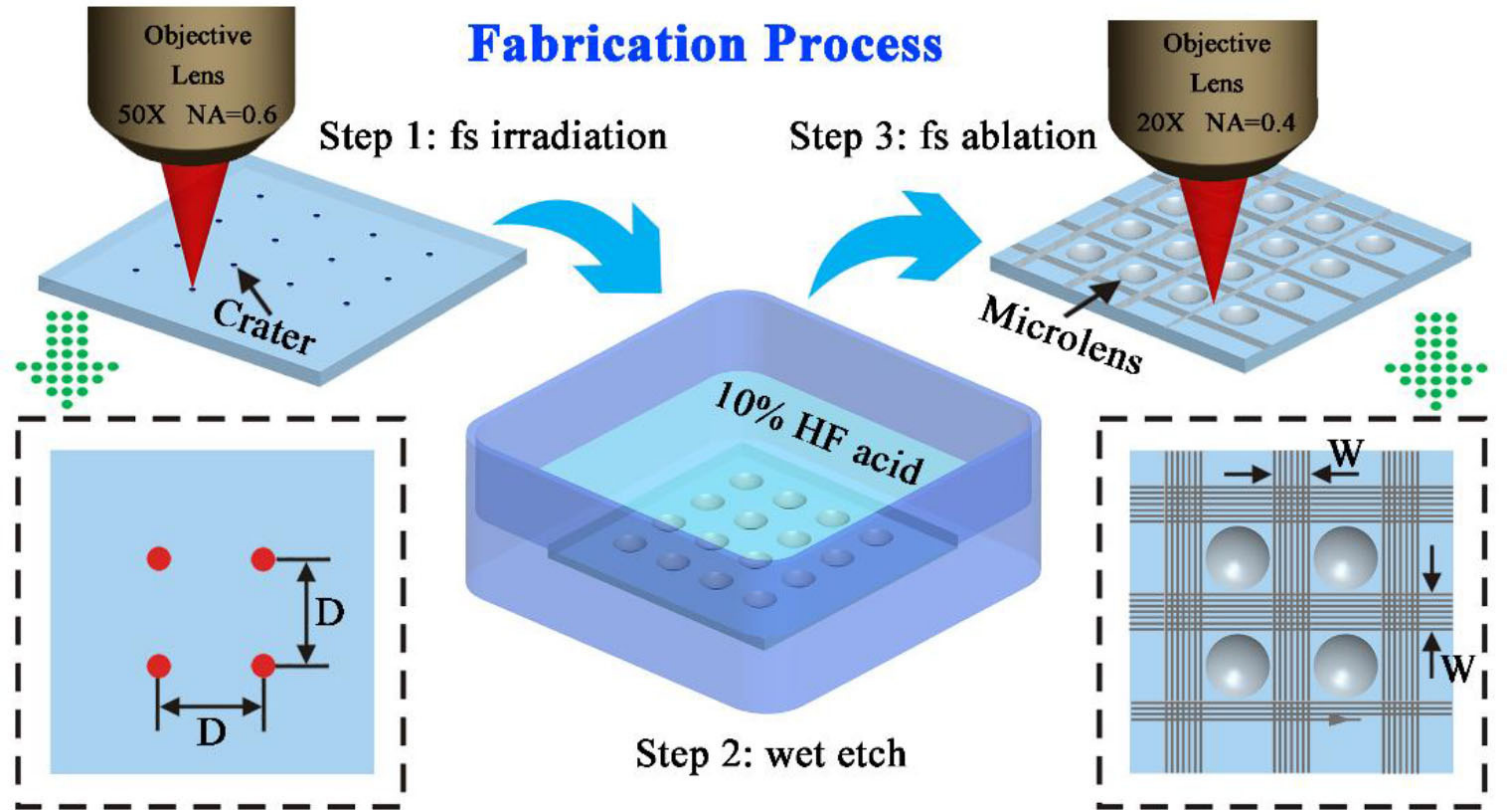
The schematic illustration of the fabrication process of the anti-oil MLA on K9 glass substrate. The lower-left image is the top view of the fs irradiation process. The lower-right image is the top view of the fs ablation rough microstructures.

Figure 1 depicts the fabrication process of the underwater anti-oil MLA. The whole fabrication process can be divided into three steps: femtosecond laser (fs) irradiation, chemical wet etch, and fs ablating surrounding criss-cross reticular rough structure.

### Femtosecond Laser Beam Irradiation

The femtosecond laser beam (pulse duration: 50 fs; central wavelength: 800 nm; reputation rate: 1 kHz) was generated from a regenerative amplified Ti: sapphire laser system (Coherent Libra-usp-he). The laser beam was focused by an objective lens (Nikon, 50 ×, NA = 0.6) on a widely used engineering material K9 glass substrate (10 × 10 × 1.5 mm) which was fixed on a computer program controlled *x-y-z* translation stage, as shown in step 1 of Figure 1. The glass surface was irradiated by fs laser via the typical point-by-point manner, forming an array of laser modification region. Each focal point was irradiated for 400 ms. The laser power which has an important influence on the size of the microlens is controlled by an attenuator. The distance (*D*) between the adjacent irradiating spots was precisely controlled by the computer program.

### Chemical Wet Etch

After the formation of uniform craters array by fs irradiation, the sample was further immersed into 10% hydrofluoric acid for 100 min, assisting with an ultrasonic water bath (40 kHz) at 25°C (step 2 of Figure 1). During the etch process, a single crater would gradually become a concave microlens shape. Next, the sample was ultrasonically cleaned with ethanol and deionized water for 5 min, respectively.

### Fs Ablation of Surrounding Grid-Patterned Rough Structure

As shown in step 3 of Figure 1. The sample was fixed on the stage and an objective lens (Nikon, 20 ×, NA = 0.4) was used to focus the laser beam (laser power: 20 mW). The focused laser was used to ablate the selective area which is between the adjacent of microlenses. The line-by-line scanning method was used to fabricate the rough structures. The laser scanning speed was set at 4,000 μm/s and the distance of adjacent laser scanning lines was set at 4 μm. The width of the laser ablation area between the adjacent microlenses was considered as *W* as shown in Figure 1.

### Sample Characterization

The morphology of the as-prepared sample was observed by a Quanta FEG 250 scanning electron microscope (SEM, FEI, America) and an OLS4000 laser confocal scanning microscope (LCSM, Olympus, Japan). The optical performance was investigated by an optical observation system that contains a commercial microscope (Nikon, 10 ×, NA = 0.3) and a charge-coupled device (CCD). The oil contact and sliding angles of a liquid droplet on the sample surface were measured by a JC2000D Contact-angle System (Powereach, China). 1, 2-Dichloroethane droplet (9 μL) was used as the probe oil. Underwater anti-oil performance of the as-prepared sample surface was observed by a wide-field microscope (Nikon, SMZ 745T). The self-cleaning function was demonstrated by immersing a sample that was pre-polluted with sesame oil in water.

## Results and Discussion

**Figure 2:**
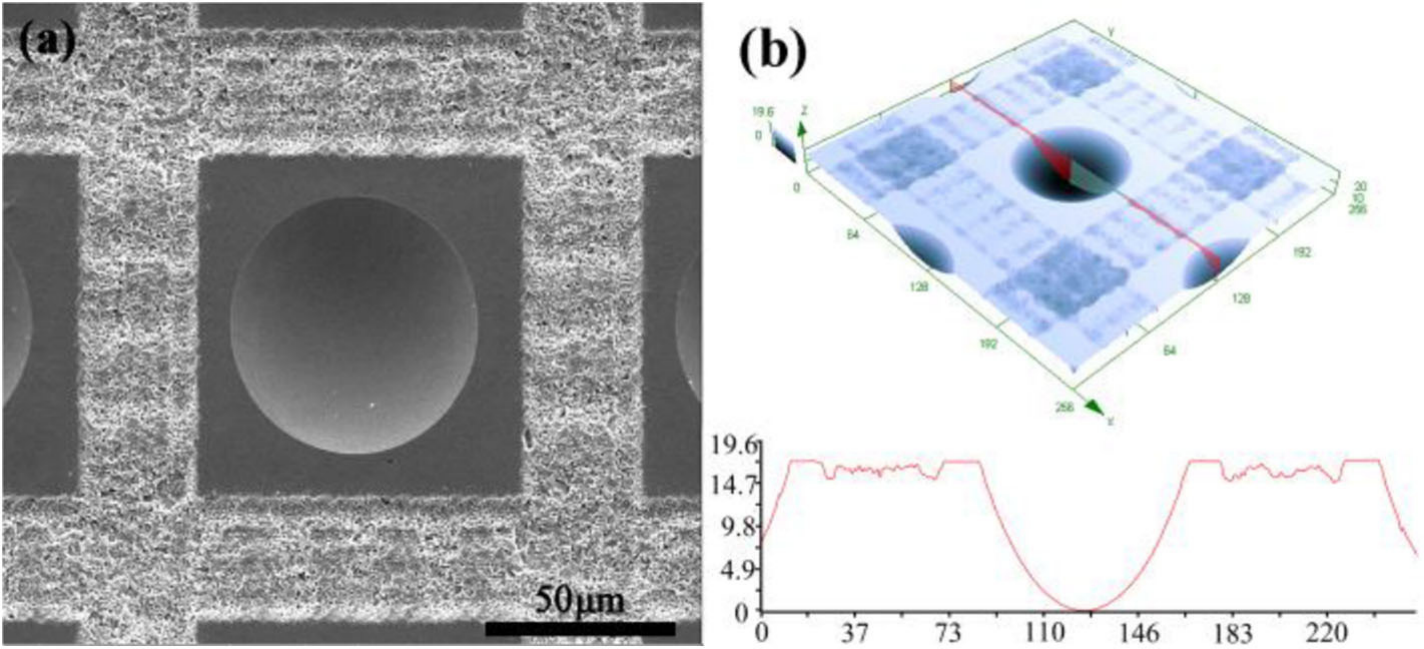
**(a)** SEM image of as-prepared microlens. **(b)** 3D morphology and cross-sectional profile of as-prepared microlens.

Figure 2 depicts the morphology of the as-prepared microlens. Here, the power of each femtosecond laser pulse was set at 3 μJ and exposure time of 400 ms. After etching 100 s in 10% hydrofluoric acid, the microlenses with excellent surface quality were successfully obtained as demonstrated in Figure 2A. The surface roughness of the microlens was also investigated at the bottom area of the microlens of 20 μm ^*^ 20 μm with an average roughness of 19.32 nm via an atomic force microscope. As shown in Figure 2B, the diameter, D, and sag height, h, of the microlenses were, respectively, measured for 59.1 ± 0.5 μm and 11.9 ± 0.5 μm. The focal length, R, and the numerical aperture, NA, can be calculated as 94.5 ± 0.5 μm and 0.31, respectively, with the refractive index of 1.45 for K9 glass (Hao et al., 2012). When the tightly focused femtosecond laser irradiated on the silica glass, strong non-linear absorption and ionization occur which transfer the optical energy absorbed by the electrons to the lattice and result in an extreme physical field with ultra-high temperature and pressure. As a consequence, coulomb microexplosion, supercontinuum and strong coupling between laser radiation and highly excited silica will take place and the focal region is surrounded by a shell of phase or structural modification. The modifications arise the decrease of the average Si–O–Si bond angle of SiO_4_ tetrahedrons and the increase of the reactivity of oxygen. Such configuration deformation could be considered as the Lewis base, which is more chemically active in reactions with acids than unmodified areas (Marcinkevicius et al., 2001; Gattass and Mazur, 2008). The chemical equation could be described as: SiO_2_+6HF < H_2_[SiF_6_]+2H_2_O. What's more, the femtosecond laser-induced nano-ripple structures in the modified region could increase the area of the acid etching and subsequently polished out by this etching process. The spherical radial distribution of the laser modified region and the amorphous properties of glass endows the fabricated structure as a microlens. The advances of the femtosecond laser enhanced chemical isotropic etching method is that the size of the microlens could be flexibly controlled via laser power, laser exposure time, chemical acid concentration and etching time, etc.

**Figure 3:**
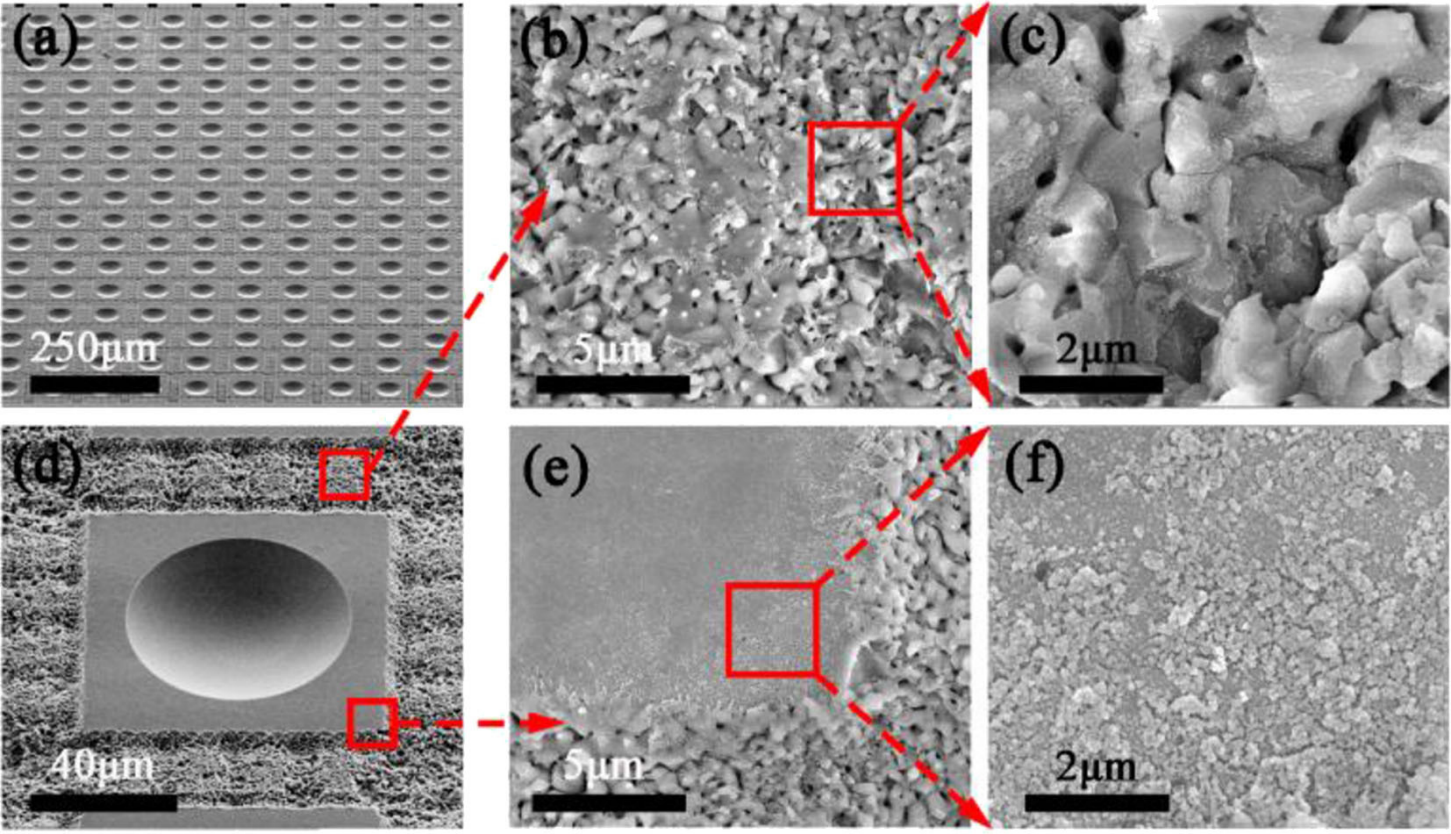
SEM images of **(a)** an as-prepared sample and **(d)** a microlens and surrounding rough structures with a 45° viewing angle. SEM images of **(b,c)** the criss-cross reticular rough structures and **(e,f)** the non-ablation area between the microlens and surrounding rough structures.

It is well-known that the wettability of various materials can be changed by creating a hierarchal micro/nano scale rough structure. Figures 3A,D depict the SEM images of the as-prepared sample which contains microlenses and laser scanning criss-cross reticular rough structures. Each microlens was arranged at the geometrical center of the non-ablation square area, which seemed as an electronic chip-like unit. The chip-like unit was a rectangular arrangement with surrounding criss-cross reticular rough structures. D and W as illustrated in Figure 1 were, respectively, set 120 and 24 μm. Figures 3B,C show the SEM images of a chip-like unit and surrounding criss-cross reticular rough structures which were formed by the laser pulse irradiation. Such special rough structures were covered with hundreds of nanometers stalactiform structures. The non-ablation area between the microlenses and rough structures were covered by abundant irregular sputtering particles at tens of nanometers (Figures 3E,F). When the femtosecond laser beam irradiation on the surface of the glass substrate, the ablated materials would be removed during the interaction between the femtosecond laser and glass materials. Besides, tens of thousands of nanoparticles would be sputtered on the surface of the sample by an explosive shock wave. These nanoparticles would redeposit and subsequently form a recast layer on the surface of the glass substrate.

**Figure 4:**
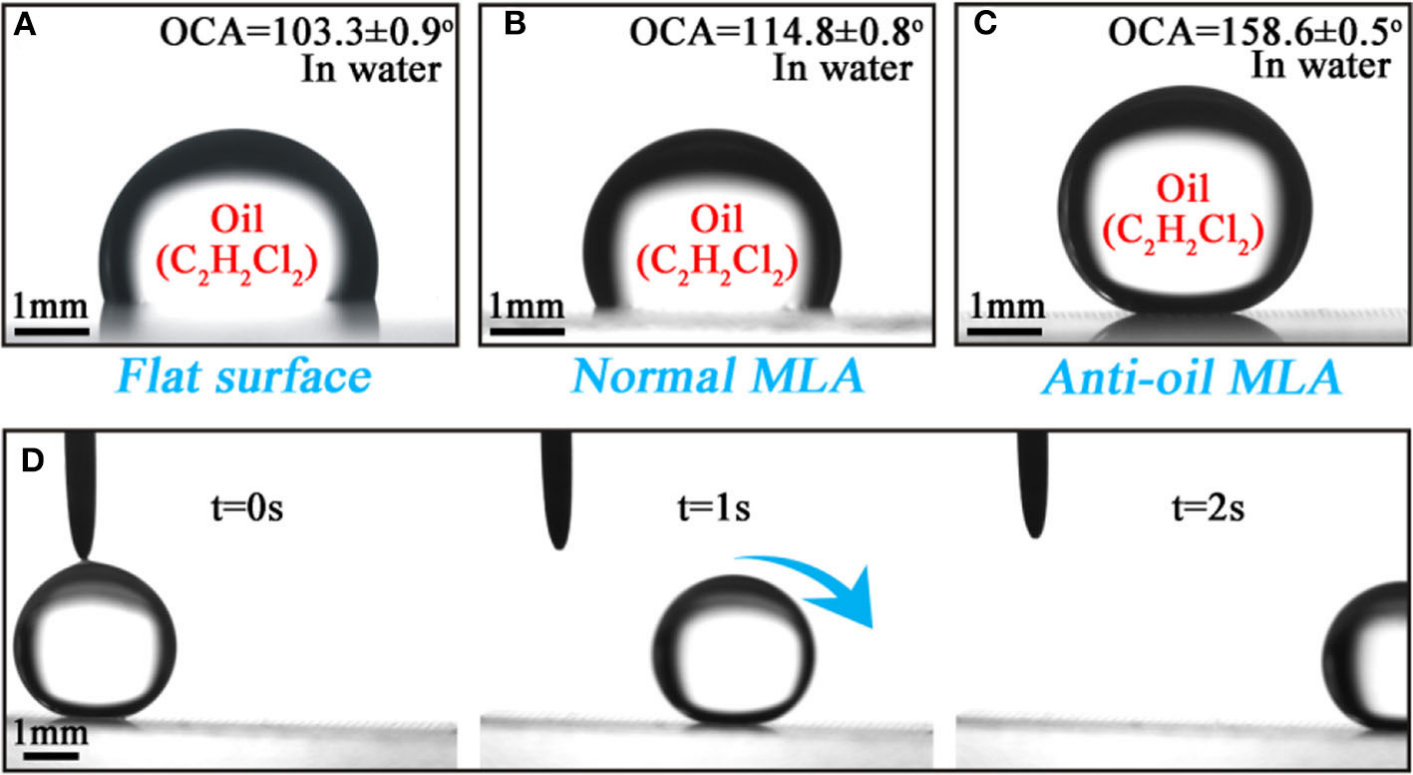
Wettability of the as-prepared samples. **(a–c)** Shapes of a 1, 2-Dichloroethane droplet (9 μL) on the **(a)** flat surface, **(b)** normal MLA surface, and **(c)** anti-oil MLA surface in water, respectively. **(d)** A series of snapshot of a 1, 2-Dichloroethane droplet rolling off on a 1° tilted as-prepared sample.

The wettability of the flat surface, normal MLA, and anti-oil MLA were, respectively, investigated and the results were exhibited in Figure 4. The oil contact angle (OCA) and oil sliding angle (OSA) of the above samples were introduced to represent their wettability. An untreated flat K9 glass substrate was immersed in water. An oil droplet was dropped on the surface of the sample which shown the intrinsic OCA of 103.3 ± 0.9° (Figure 4A). An oil droplet was dropped on a normal MLA which was fabricated by femtosecond laser induced chemical etching. The OCA was measured for 114.8 ± 0.8° (Figure 3B). This phenomenon was caused by the tens of micrometers concave structures which were occupied by the oil. As a consequence, the contact area between the oil and the surface were larger than that of the flat surface which would lead to a higher OCA. On the contrary, as shown in Figure 4C, when an anti-oil MLA dipped into water, the oil droplet almost keeps as a spherical shape which shows an underwater superoleophobicity with an OCA of 158.6 ± 0.5°. Moreover, the oil droplet would instantly roll off when the as-prepared sample was tilted for 1° (OSA), which reveals the ultralow oil-adhesion of the as-prepared sample (Figure 3D).

**Figure 5:**
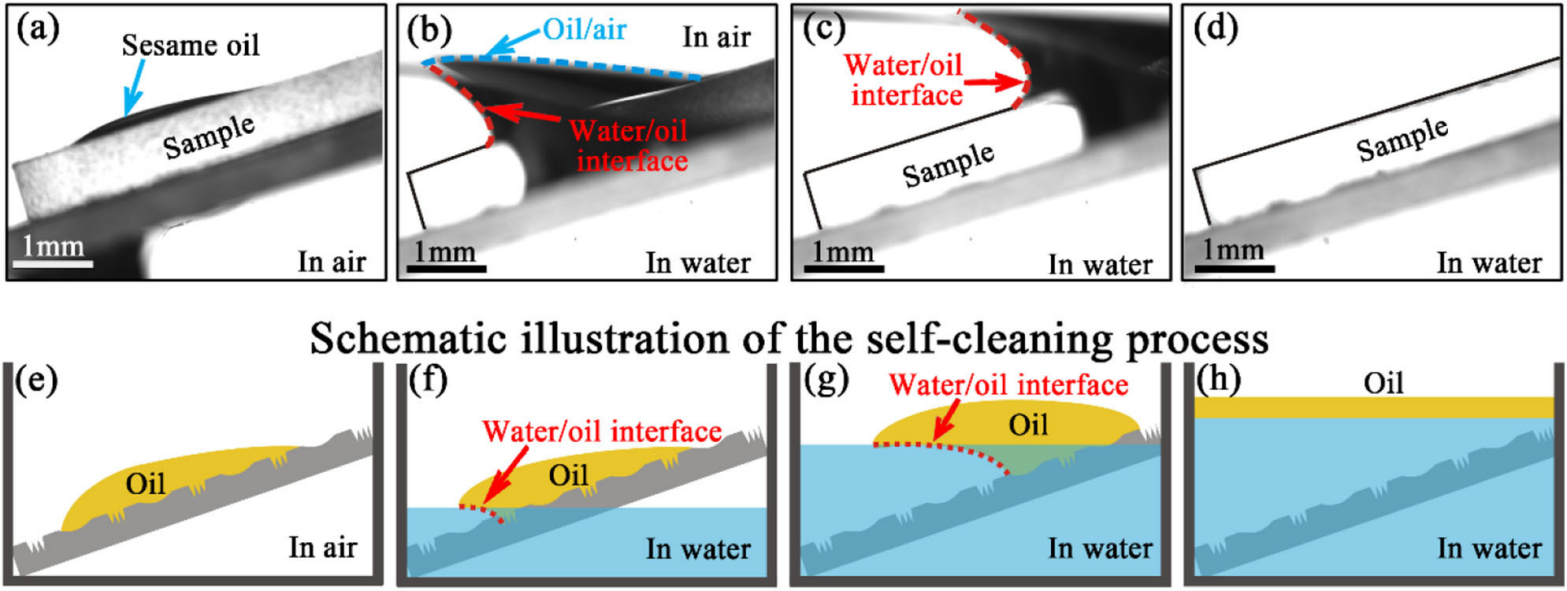
Time series snapshots of the anti-oil and self-cleaning process. **(a)** A sesame oil droplet dropped onto the surface of the as-prepared sample. **(b,c)** The oil was removed with the surface gradually immersed in water. **(d)** The clean surface without any remaining oil. **(e–h)** Schematic illustration of the self-cleaning ability of the anti-oil MLA.

The rough structure surface which exhibits ultralow oil adhesion and underwater superoleophobicity endows the surface with anti-oil and self-cleaning function. Figures 5A–D show the time series snapshots of the experiment. As shown in Figure 4A, a common sesame oil droplet was dropped onto the surface of the as-prepared sample which was titled for 20°. Figures 5B,C show the snapshots of oil droplet removed by the water. The surface of the sample would be cleaned without any residue oil which is shown in Figure 5D. Figures 5E–H show the schematic illustration of the self-cleaning function of the as-prepared sample. The oil droplet immediately spread out while dropped onto the surface of the sample (Figure 5E). The interspace of the rough structures will be occupied by the water instead of the oil once the surface immersed in water. Due to the ultralow oil adhesion of the as-prepared sample. The oil droplet would be gradually pushed away and removed from the surface. With the further surface of the sample immersed in water, the water/oil interface moves from bottom left to the top right (Figures 5F,G). When the sample completely immersed in water, the oil was removed without any residues and float on the surface of the water (Figure 5H). The as-prepared sample exhibits superb anti-oil and self-cleaning functions which will reduce the complicated cleaning process of the optical devices and prolong the service life.

**Figure 6:**
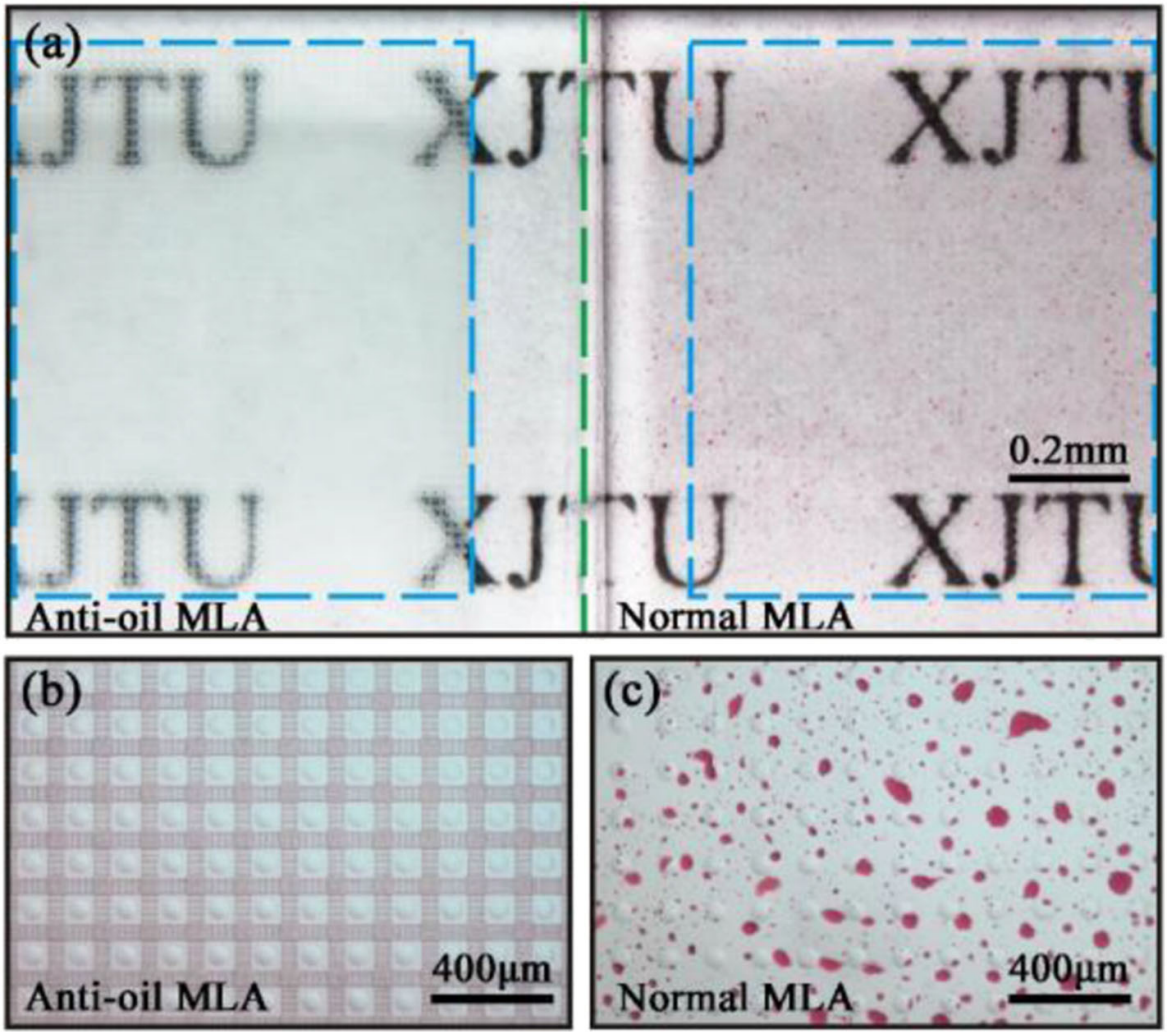
**(a)** Oil resistance of the anti-oil MLA compared with the normal MLA. **(b,c)** Magnified images of normal MLA and anti-oil MLA. The green imaginary line represents the boundary of two samples, and the rectangle blue imaginary lines represent the anti-oil MLA and normal MLA.

In order to investigate the oil resistance of the fabricated anti-oil MLA. A normal MLA sample was used to make a comparison with an as-prepared anti-oil MLA. These samples were immersed in water which contained petroleum ether (dyed with Sudan III, red color) for 1 min. Then the samples were taken out and observed by a wide-field microscope. Figure 6A depicts the image of the anti-oil MLA sample and the normal MLA sample after contamination by petroleum ether solution. The magnified images are, respectively, shown in Figures 6B,C. The anti-oil MLA was sufficiently clean without any oil rest on the surface (Figure 6B). On the contrary, the normal MLA was contaminated by petroleum ether. A mass of oil adhered to the surface of the MLA which cannot be easily cleaned (Figure 6C). Therefore, the fabricated resultant anti-oil MLA exhibited excellent oil resistance.

**Figure 7:**
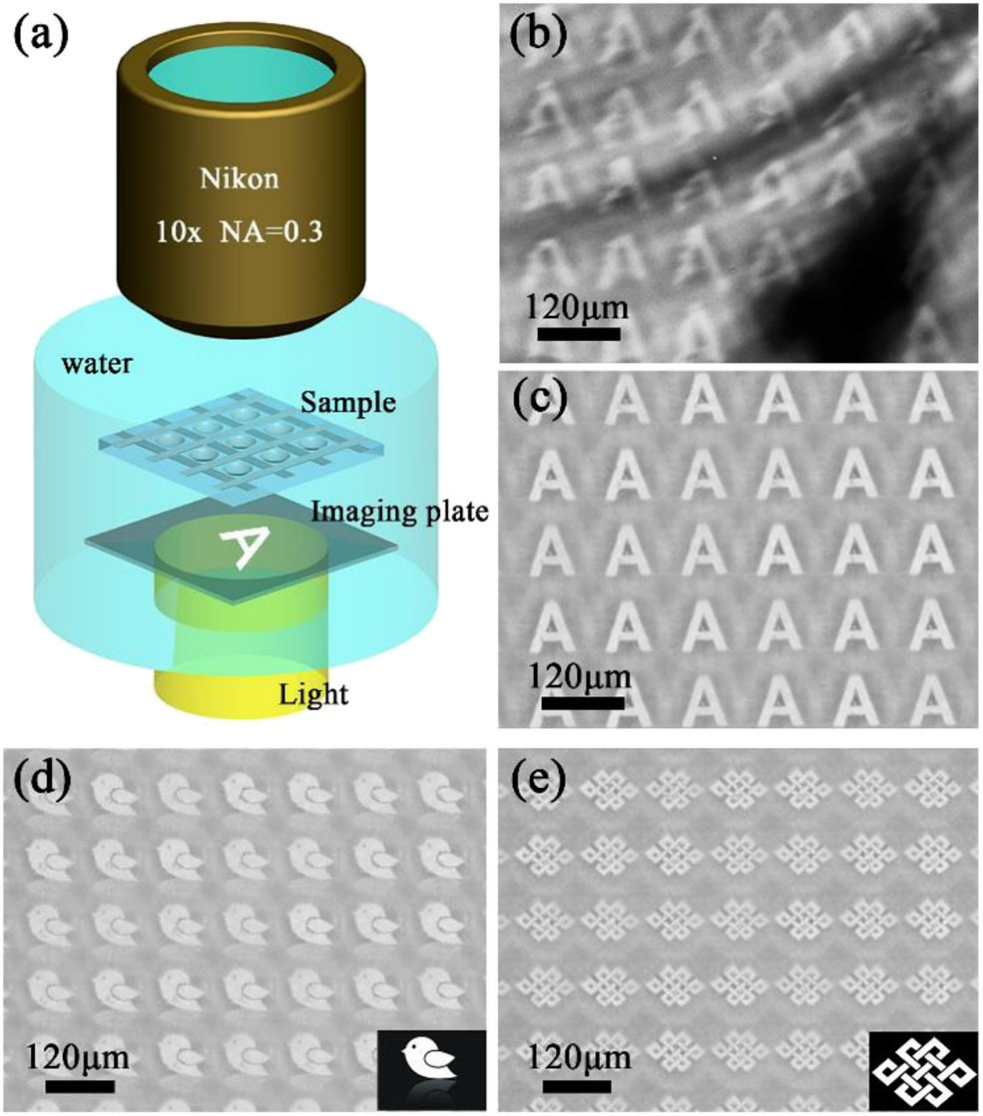
**(a)** The schematic illustration of the underwater optical observation system. The virtual images produced by **(b)** normal MLA and **(c)** anti-oil MLA after oil contamination. The virtual images of **(d)** a bird with inverted image and **(e)** a Chinese knot.

Then the imaging property of the normal MLA and anti-oil MLA after oil contamination were investigated by an underwater optical observation system (Figure 7A). The as-prepared sample and an imaging mask plate with a transparent letter “A” were immersed in water between the objective lens and light source to simulate the underwater imaging. The imaging results of the anti-oil MLA and normal MLA after oil contamination were shown in Figures 7B,C, respectively. The normal MLA was vague, seriously distortion, and even cannot form an image (Figure 7B). After oil contamination, the surface of the normal microlenses was adhered by oil pollutants, due to the different refractive index of the oil and glass, the images would be seriously distortion. Even worse, oil droplets would stick to other substances in water. As a consequence, these mixture pollutants would impede microlenses imaging. However, due to the anti-oil and self-cleaning capacity, oils and pollutants cannot contaminate the sample. The image letters “A” of anti-oil MLA was clearly and uniformly (Figure 7C). Moreover, different patterns of imaging results of anti-oil MLA were shown in Figures 7D,E, which shows the excellent imaging property of the anti-oil MLA. During the underwater detection, the interspace of rough structures will fill with water once the sample immersed in water. Due to the similar refractive index of the water and the K9 glass, the small refraction and scattering occur while light passes through the interface between these two materials, which endows the sample a good transparency during underwater detection.

**Figure 8:**
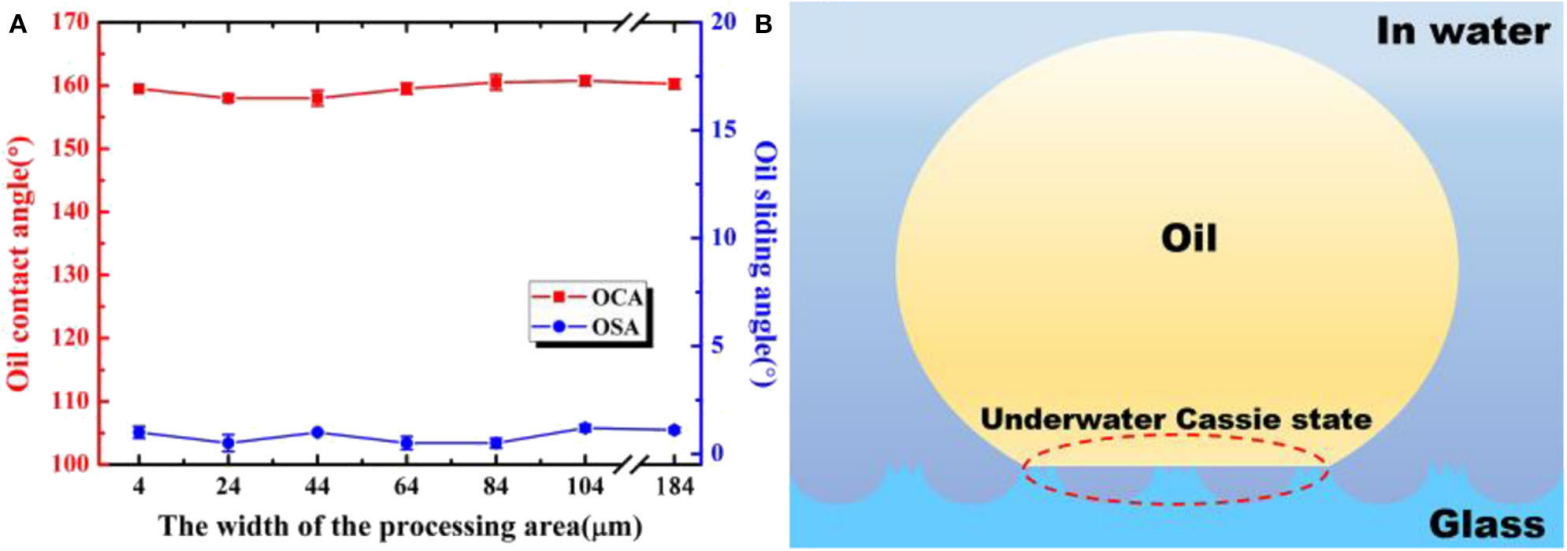
**(a)** OCAs and OSAs of underwater oil droplets on samples with different widths of the processing area. **(b)** Illustration of the wetting state of an oil droplet on as-prepared structure in water.

The width of the laser processing area (*W*) which shown in step 3 of Figure 2 is an important processing parameter that would influence the ratio of the anti-oil microlens unit and surrounding criss-cross reticular rough structures. Figure 8A illustrates the OCA and OSA underwater wettability with different W. During this section, the size of the microlenses and untreated rectangle area remains the same. It is obvious that the OCAs of all samples remain higher than 155° which shows underwater superoleophobicity with the W increases from 4 to 184 μm. Meanwhile, the OSAs stay lower than 2° which presents underwater ultra-low oil-adhesion of all samples. Almost the same OCAs and OSAs of different W indicate that the variation of the ratio between anti-oil microlens unit and surrounding criss-cross reticular rough structures not influence the wettability of the samples. In fact, the rough structures of the anti-oil MLA include two main topographies. One is the surrounding criss-cross reticular hierarchal micro/nano rough structures, and the other is the recast layer of the abundant irregular sputtering nanoparticles at the non-ablation area, as shown in Figure 3. The contact mechanism of oil droplets with these two kinds of rough structures and concave microlens structure can be illustrated by an underwater Cassie state, as shown in Figure 8B. While the sample immersed in water, the water immediately trapped in the interspace between rough structures and concave microlens structure which would contribute to the underwater superoleophobicity. Therefore, the trapped water would form a layer which impedes the contact of the oil droplet with the sample. As a matter of fact, the oil droplet only contacted with the peak of the rough structures and rested in an oil-water-solid three-phase system. The trapped water reduced the contact area between oil droplet and surface of the sample, which would lead to an ultralow oil-adhesion of the as-prepared sample.

**Figure 9:**
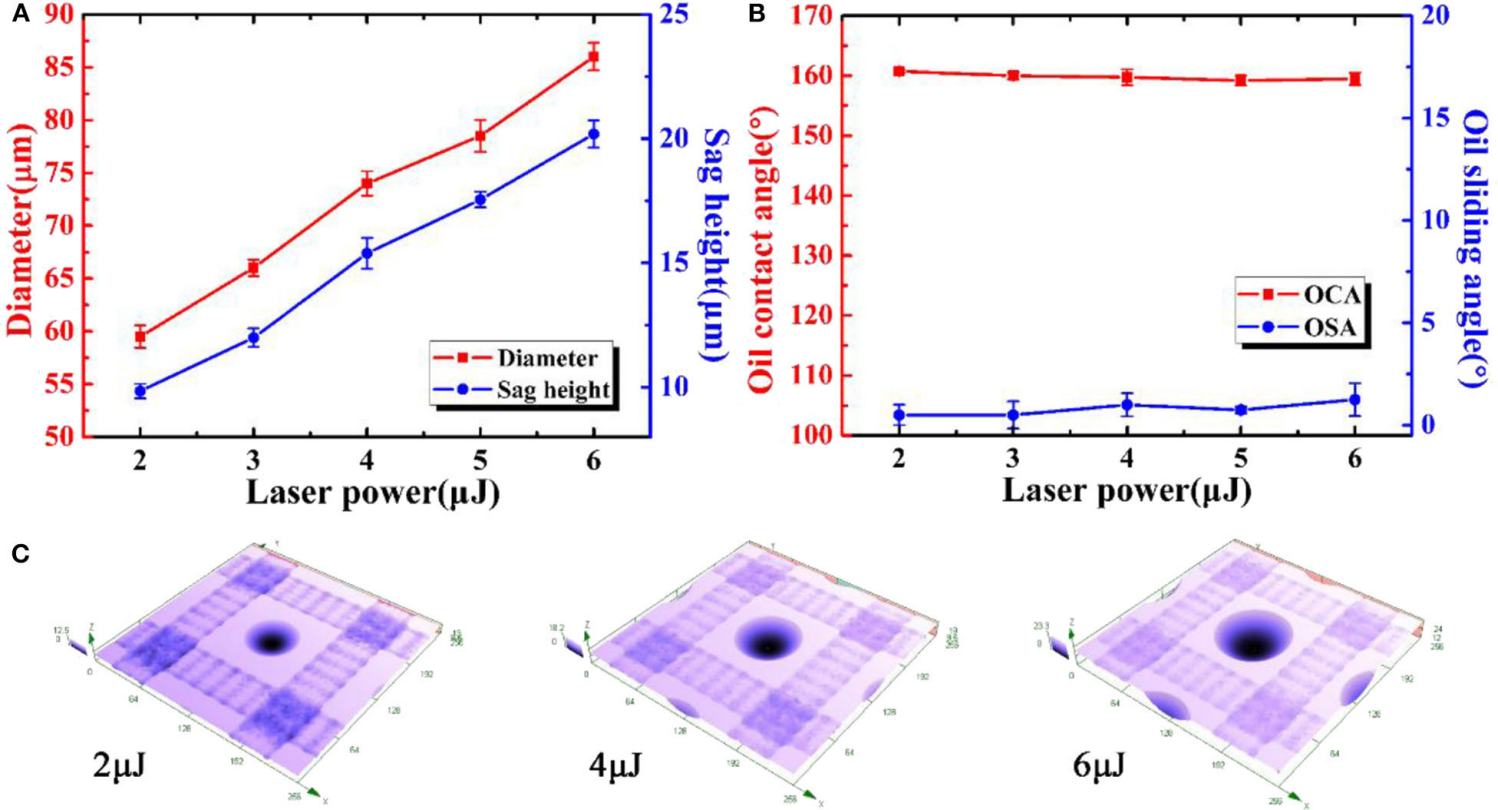
**(a)** Relationship between the laser power of the irradiation process and the sizes of the microlenses. **(b)** OCAs and OSAs of underwater oil droplets on the anti-oil MLA with different laser power of the irradiation process. **(c)** 3D morphologies of the laser power during irradiation process for 2, 4, and 6μJ, respectively.

Different sizes of the microlenses can be obtained by simply varying the laser power during laser pulse irradiation process. In order to investigate the influence of the laser power of the irradiation process on the sizes of the microlens and wettability of the sample, *D* and *W* of all samples in this section were set at 160 and 44 μm, respectively. Figure 9A illustrates the influence of the irradiation laser power on the diameter and the sag height of the microlens after chemical treating for 100 min. The diameter and the sag height of the microlens increase with the rise of the laser power which can be considered as a linear relationship. As shown in Figure 9B, with the increasing of the laser power of the irradiation process, the OCAs and the OSAs mainly remain unchanged for about 160 ± 2° and 1.5 ± 1°, respectively. The results indicate the size of the microlens does not affect the underwater oil repellence. Figure 9C shows the three-dimensional (3D) morphologies of the laser power during the irradiation process for 2, 4, and 6 μJ, respectively. As shown, the size of microlens will increase with the rise of laser irradiation power.

## Conclusions

In conclusion, a novel anti-oil MLA was rapidly fabricated on a glass substrate by the above demonstrated femtosecond laser-induced phase and structural modification and chemical isotropic etching processes. Through the ingenious combination of ultra-smooth microlenses and micro/nano rough structures, both optical imaging and anti-oil capabilities are produced. The smooth MLA can be used for underwater imaging while the hierarchical micro/nano rough structures give the sample underwater anti-oil ability. It has been demonstrated that the anti-oil MLA will stay clean when it is immersed in oil solution. After the oil contamination, the sample can be easily washed clean with water. Therefore, it can be considered an effective oil-repellent and self-cleaning MLA. In addition, different sizes of the microlenses and a wide variation of the widths of the rough structures between adjacent microlenses were investigated. As a results, they all showed underwater superoleophobicity and ultralow oil-adhesion. We believe the femtosecond laser hybrid process resultant anti-oil MLA will be widely applied in bioscience research, ocean exploration, and underwater optical detection.

## Data Availability Statement

The original contributions presented in the study are included in the article/supplementary material, further inquiries can be directed to the corresponding author/s.

## Author Contributions

HB directed and supervised the research and wrote the manuscript. JL and YW performed the experiments. ML contributed toward signicant discussions and revised the paper. All authors contributed to the article and approved the submitted version.

## Conflict of Interest

The authors declare that the research was conducted in the absence of any commercial or financial relationships that could be construed as a potential conflict of interest.
